# Application of the dual-luciferase reporter assay to the analysis of promoter activity in Zebrafish embryos

**DOI:** 10.1186/1472-6750-8-81

**Published:** 2008-10-27

**Authors:** Francisca Alcaraz-Pérez, Victoriano Mulero, María L Cayuela

**Affiliations:** 1 Research Unit, Department of Surgery, University Hospital "Virgen de la Arrixaca", 30120 Murcia, Spain; 2 Department of Cell Biology and Histology, Faculty of Biology, University of Murcia, 30100 Murcia. Spain

## Abstract

**Background:**

The dual-luciferase assay has been widely used in cell lines to determine rapidly but accurately the activity of a given promoter. Although this strategy has proved very useful, it does not allow the promoter and gene function to be analyzed in the context of the whole organism.

**Results:**

Here, we present a rapid and sensitive assay based on the classical dual-luciferase reporter technique which can be used as a new tool to characterize the minimum promoter region of a gene as well as the *in vivo *response of inducible promoters to different stimuli. We illustrate the usefulness of this system for studying both constitutive (telomerase) and inducible (NF-κB-dependent) promoters. The flexibility of this assay is demonstrated by induction of the NF-κB-dependent promoters using simultaneous microinjection of different pathogen-associated molecular patterns as well as with the use of morpholino-gene mediated knockdown.

**Conclusion:**

This assay has several advantages compared with the classical *in vitro *(cell lines) and *in vivo *(transgenic mice) approaches. Among others, the assay allows a rapid and quantitative measurement of the effects of particular genes or drugs in a given promoter in the context of a whole organism and it can also be used in high throughput screening experiments.

## Background

The zebrafish has been established as an excellent model for studying any biological process. This organism possesses many advantages including ease of experimentation, optical clarity, drug administration, amenability to *in vivo *manipulation and feasibility of reverse and forward genetic approaches. The fish reach sexual maturity in only 3 to 4 months, and adult females are capable of producing 100 to 200 eggs weekly. Many thousands of animals can be kept in a fish facility requiring much less space than mice or other mammals, and hence the zebrafish is regarded as a cost-effective experimental vertebrate model for large-scale genetic screening [1]. Furthermore, the high degree of homology between the zebrafish genome and that of humans makes such discoveries especially pertinent to human disease and development [2,3].

Morpholino antisense oligonucleotides (MO) have been widely used to inhibit gene function in zebrafish embryos [4-7] and are usually used as sequence-specific translation-blocking or splicing-blocking agents. Recently, a quantitative assessment of the knockdown efficiency of morpholinos has been performed in zebrafish embryos and its effectiveness proved [8]. Furthermore, microinjection of DNA constructs into single-cell fertilized zebrafish embryos has also proven successful in the generation of transgenic zebrafish. The widespread use of fluorescent proteins in mammalian systems has been successfully adapted for use in zebrafish, which are well-suited to the use of fluorescence because of their optical clarity and external development. By linking a fluorescent protein such as enhanced green fluorescent protein (eGFP) or *Discosoma sp*. red fluorescent protein (DsRed) to a gene or promoter of interest, expression can be easily visualized in living animals [9].

The dual luciferase assay has been widely used in cell lines to determine rapidly and accurately the activity of a given promoter. Although this strategy has been very useful, it does not allow analysis of the promoter and gene function in the context of the whole organism. To overcome these limitations, we have developed a protocol based on the dual luciferase system in zebrafish embryos. We illustrate the usefulness of this system for studying the promoter of telomerase, a key enzyme in the fields of cancer, stem cells and aging [10], and a NF-κB-dependent promoter, a master regulator of the immune response [11]. The luciferase reporter DNA plasmids were injected into zebrafish embryos at the one-cell developmental stage, together with MO or the expression constructs of interest, and the luciferase activity was determined in the time frame of MO activity (24–48 h later). In addition, the flexibility of this assay is also illustrated by activation of the NF-κB-dependent promoters by simultaneous microinjection of different pathogen-associated molecular patterns (PAMPs).

The protocol presented here provides details of how to apply the dual-luciferase assay to determining the activity of constitutive and inducible promoters in zebrafish embryos. This approach involves three steps: (1) cloning the promoter of interest in the firefly luciferase reporter construct, (2) microinjecting the embryos with this construct together with the appropriate *Renilla *luciferase reporter and (3) measuring the promoter activity with the dual luciferase system in whole embryo extracts.

## Results

**Figure 1:**
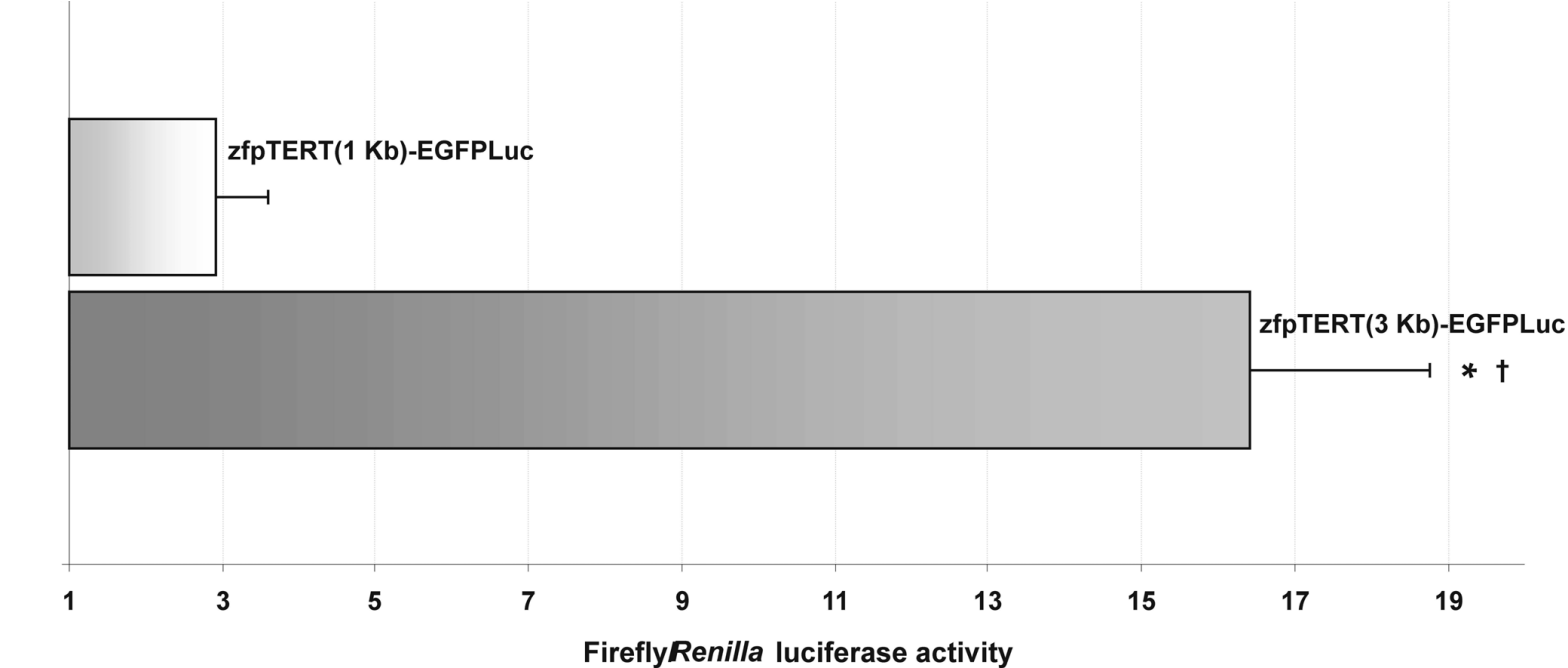
**Analysis of zfTERT promoter activity in whole zebrafish embryos**. Zebrafish one to eight-cell embryos were microinjected with plessEGFPLuc, zfpTERT(1 Kb)-EGFPLuc or zfpTERT(3 Kb)-EGFPLuc and the pRL-CMV (10:1) reporter vectors. Twenty-four hours after microinjection, the firefly and Renilla luciferase activity was measured using the Dual-Luciferase Reporter Assay System. The results are expressed as the mean ± S.E. of normalized luciferase activity relative to plessEGFPLuc injected embryos. *p < 0.05 vs. plessEGFPLuc. ^†^p < 0.05 vs. zfpTERT(1 Kb)-EGFPLuc.

The protocol presented here should result in very sensitive and accurate measurement of promoter activity and analysis of gene function in the context of the whole organism, which represents an important advantage over traditional measurement in cell lines. We first illustrated the usefulness of our protocol to analyze the promoter activity of zebrafish telomerase-reverse transcriptase (zfTERT) (Fig. 1). At 24 h post-injection (hpi), the 3 Kb fragment upstream of the zfTERT coding sequence was able to drive the expression of the firefly luciferase reporter while the 1 Kb fragment failed to significantly increased the basal expression. Therefore, the relative promoter activity of each fragment could be quantitatively determined.

**Figure 2:**
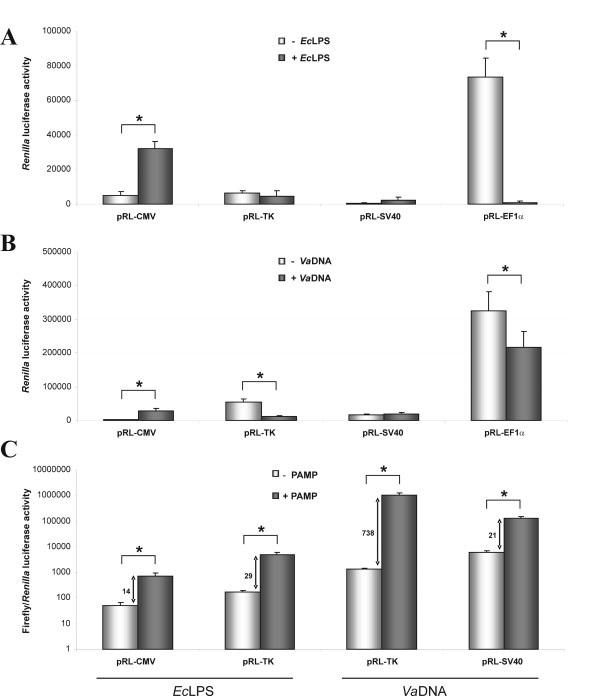
**Optimization of the different *Renilla *luciferase reporter constructs**. Zebrafish one to eight-cell embryos were microinjected with 30 ng EcLPS **(A, C) **or 6.5 ng *Va*DNA **(B, C) **and NF-κB::Luc together with the pRL-CMV, pRL-TK, pRL-SV40 or pRL-EF1α (10:1) reporter vectors. Twenty-four hours after microinjection, the firefly and Renilla luciferase activity was measured using the Dual-Luciferase Reporter Assay System. The results are presented as the *Renilla *luciferase activity (A, B) or as the normalized luciferase activity (firefly/*Renilla*) (C). Each bar represents the mean ± S.E. of ten replicate samples and the data are representative of three independent experiments. The asterisk denotes statistically significant differences between the indicated samples.

The critical step in the protocol presented here is the correct choice of the promoter used for normalization. The cytomegalovirus (CMV) immediate-early promoter is a strong promoter used for both the *in vitro *and *in vivo *expression of proteins in signal transduction and gene therapy studies. However, CMV activity is induced by external stimuli such as endotoxin from Gram-negative bacteria (lipopolysaccharide, LPS), cytokines and phorbol esters [12]. Therefore, for the study of NF-κB activation, we first have studied the effects of bacterial LPS and DNA in the expression of several *Renilla *luciferase reporter constructs. Among the four candidates tested, the CMV promoter was strongly induced by both PAMPs, i.e. *Escherichia coli *LPS (*Ec*LPS) and *Vibrio anguillarum *DNA (*Va*DNA), whereas the translation elongation factor EF1α promoter was inhibited by *Ec*LPS and, to some extent, by *Va*DNA. In contrast, the herpes simplex virus thymidine kinase (TK) promoter and the early SV40 enhancer/promoter region showed a more constant expression (Fig. 2A and 2B) and were therefore selected for further studies. Figure 2C illustrates the profound effects of the plasmid used for normalization in the measurement of the induction of NF-κB. When using *Ec*LPS, a 29 vs. 14 fold induction of NF-κB activity was obtained with the TK and the CMV promoters, respectively (Fig. 2C), indicating that induction of CMV by *Ec*LPS resulted in the underestimation of the NF-κB activation by this PAMP. Similarly, 21 vs. 738 fold induction of NF-κB was observed with *Va*DNA when using the SV40 and the TK promoters, respectively (Fig. 2C), indicating that the inhibition of the TK promoter by *Va*DNA resulted in the overestimation of the NF-κB induced by this PAMP.

**Figure 3:**
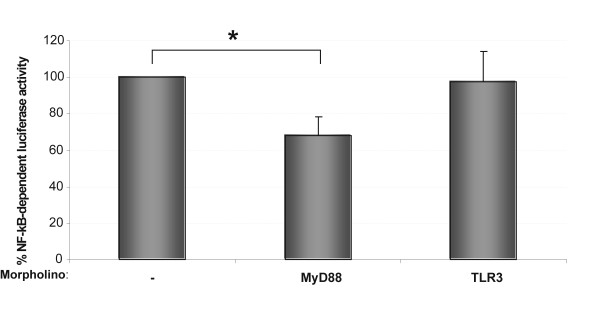
**An example of the usefulness of MO-gene mediated knockdown in combination with the dual-luciferase assay for the analysis of the NF-κB signaling pathway**. Zebrafish one to eight-cell embryos were microinjected with 6.5 ng *Va*DNA together with the firefly and *Renilla *reporter vectors as indicated in Legend to Figure 2 alone or in combination with 4 ng of the indicated morpholinos. Twenty-four hours after microinjection, activation of the NF-κB activation was measured using the Dual-Luciferase Reporter Assay System. The results are expressed as normalized luciferase activity relative to control embryos not injected with MOs. Each bar represents the mean ± S.E. of ten replicate samples and the data are representative of three independent experiments. The asterisk denotes statistically significant differences between the indicated samples.

We finally validated the usefulness of this technique for studying a gene of interest by using MO-gene mediated knockdown. Figure 3 illustrates an example of the inhibition of the NF-κB activation triggered by *Va*DNA using a translation-blocking MO against MyD88 [13], an adaptor protein involved in the downstream signalling following the engagement of bacterial DNA by its cognate receptor (TLR9) [14]. The results showed that injection of the MO against MyD88 resulted in a significant inhibition (> 30%) of the NF-κB activation induced by bacterial DNA, while injection of a MO directed against TLR3, which is involved in the recognition of double-stranded RNA [15], failed to affect the NF-κB activation induced by bacterial DNA (Fig. 3).

## Discussion

The protocol presented here provides details of how to apply the dual-luciferase assay to determining the activity of both constitutive and inducible promoters in zebrafish embryos. Beyond genetics and experimental tools, the strength of the zebrafish resides in the analysis of phenotype [1]. Perhaps no other organism (and certainly no vertebrate) is better suited to high-throughput phenotyping. The scale that can be achieved in zebrafish experiments is impressive by vertebrate standards. Early zebrafish embryos are less than 1 mm in diameter, allowing several embryos to fit easily in a single well of a 384-well plate. Whole organisms offer several advantages over cell lines for forward chemical genetic screens, providing information on tissue specificity, toxicity and accounting for bioavailability. Furthermore, cells are not transformed and are in their normal physiological milieu of cell-cell and cell-extracellular matrix interactions [16-18]. Use of the whole organism can also allow the screening of processes that are not easily replicated *in vitro *such as organ development. The advantages of zebrafish screening over invertebrate model organisms are their closer evolutionary relationship to humans [16-18]. Therefore, the assay described here represents a promising route to the identification and validation of novel drug targets. Analysis of the promoter of newly identified genes that underlie zebrafish disease phenotypes might lead directly to the identification of novel drug targets or genes that can correct the phenotype.

Because zebrafish development occurs *ex uterus *and they have a large number of offspring, hundreds or thousands of embryos can be injected per day and the results of this assay can be obtained within 24–48 h, although shorter time points can also be analyzed if either the mRNA coding for the gene under study or the recombinant protein are used. Although some variation was found between replicates, these can be easily avoided by the high number of technical replicates achievable. The high-fold induction of luciferase activity, together with barely detectable levels of basal expression, makes it an ideal system for the *in vivo *analysis of inducible promoters. In addition, the assay can be combined with powerful MO-gene mediated knockdown or gene over-expression to rapidly determine the functions of a particular gene (1–2 days compared with months-years needed for the generation of knockout mice). Therefore, this technique appears to be suitable for studying the activity and responses of different promoters and gene functions as well as for the validation of genetic constructs. However, this technique does not provide spatial information on gene expression and, therefore, it might be useful as a complementary technique to *in situ *hybridization and fluorescent microscopy. To avoid this limitation, all our promoters drive the expression of a fusion of eGFP and firefly luciferase, which might allow the simultaneous determination of the expression levels and the spatial localization of the promoter under analysis.

A shortcoming of the present assay, however, is the transient expression of the constructs and, therefore, only short-term responses of promoters can be studied. For example, the adaptive immune response can not be studied with the assay since it develops after several weeks. On the other hand, we have found that normalization is absolutely required for the elimination of experimental variations. As we have found that the *Renilla *luciferase plasmid used for normalization can be induced by external stimuli, the choice of the normalization plasmid is critical. Thus, the CMV immediate-early promoter, which is commonly used for normalization in both *in vitro *and *in vivo *studies, is significantly induced by external stimuli such as endotoxin (LPS) and genomic DNA from bacteria, as previously reported in cell lines with LPS, cytokines and phorbol esters [12]. However, this limitation could be easily overcome using other commercial *Renilla *luciferase reporter vectors, such as those driven by the herpes simplex virus TK promoter (pRL-TK) or the early SV40 enhancer/promoter region (pRL-SV40).

## Conclusion

We have developed a rapid and sensitive assay based on the classical dual-luciferase reporter technique which can be used as a new tool to characterize the minimum promoter region of a gene and the *in vivo *response of inducible promoters to different stimuli as well as in high throughput screening experiments. The flexibility of this assay is demonstrated by induction of the NF-κB-dependent promoters using simultaneous microinjection of different PAMPs as well as with the use of MO-gene mediated knockdown.

## Methods

### Reagents

• Firefly (*Photinus pyralis*) luciferase reporter plasmids (Table 1).

**Table 1:** Reporter plasmids used in this study

**Plasmid name**	**Purpose**	**Relevant features**
pEGFPLuc	Positive control for assaying the effectiveness of transfection	Kanamycin/neomycin marker. This reporter plasmid encodes a fusion of eGFP and luciferase from the firefly *Photinus pyralis *driven by the human CMV immediate early promoter (Clontech, Cat.# 6169-1).
plessEGFPLuc	Negative control	Kanamycin/neomycin marker. This vector was created by removing the CMV promoter of pEGFPLuc with the restriction enzymes AseI and NheI, followed by blunting of 5'-cohesive ends and autoligation.
zfpTERT(1/3 Kb)-EGFPLuc	Determination of the zfTERT promoter activity	Kanamycin marker. This vector was created by replacing the CMV promoter of pEGFPLuc with a 1 or 3 Kb-fragment of the zebrafish telomerase promoter region.
pNF-κB::Luc	Assessment of NF-κB activation	Ampicillin marker. This vector carries a the cDNA encoding the firefly (*P. pyralis*) luciferase gene placed under the control of three synthetic copies of the κB consensus of the immunoglobulin κ-chain promoter cloned in the *Bam*HI site located upstream of the conalbumin transcription start site [19].
pRL-CMV, pRL-TK and pRL-SV40	Normalization	Ampicillin marker. The pRL vectors contain the cDNA encoding Renilla luciferase cloned from the anthozoan coelenterate *Renilla reniformis *(sea pansy). Three different promoter configurations are available; CMV, TK and SV40.
pRL-EF1α	Normalization	Ampicillin marker. This vector was obtained by inserting the EF1α promoter in the pRL-null vector (Promega, Cat. # E2271).

*Note*: pEGFPLuc has been discontinued and is no longer available from Clontech; it can be obtained from the authors on request. Full sequences and maps can be found on the Clontech and Promega websites.

• Sea pansy (*Renilla reniformis*) reporter plasmids from Promega: pRL-CMV (Cat.# E2261), pRL-TK (Cat.# E2241) and/or pRL-SV40 (Cat.# E2231) (Table 1).

• Adult zebrafish.

• Egg water (60 mg/l Ocean Salt, 0.45 mM NaHCO_3_, 0.0375 mM CaCO_3_, 0.05% Methylene Blue)

• Agarose-modified Petri dish as described in the Zebrafish book .

• Phenol Red Solution (0.5% in PBS, Sigma-Aldrich).

• Buffer Tango 10× (Fermentas).

• Morpholinos 1 mM stock solution in distilled water (Gene Tools, LLC).

◈ MO-Myd88: 5'-TAGCAAAACCTCTGTTATCCAGCGA-3' [13]

◈ MO-TLR3: 5'-GTAAAAACATACCTTTAAGAGAGAG-3'

• EcLPS: LPS from *Escherichia coli *strains 0111:B4 (Cat.# L4391) or 055:B5 (Cat.# L6529) from Sigma-Aldrich.

• LPS from *E. coli *strains 0111:B4 conjugated with FITC (Cat.# F3625).

• *Va*DNA: phenol-extracted genomic DNA from *Vibrio anguillarum*.

• Dual-Luciferase Reporter Assay System (Promega, Cat.# E1910).

• Phosphate-buffered saline (PBS).

### Equipment

• Air incubator set at 28.5°C (Memmert).

• Curved-tip forceps.

• Straight-tip forceps.

• Puller (PC-10, Narishige).

• Glass Capillaries with filament (GD-1, Narishige).

• Microloader tips (0.5–10 μl, Eppendorf).

• Microinjector (IM300, Narishige).

• Stereomicroscope (MZ6, Leica).

• Pellet pestle, disposable (Sigma-Aldrich): Cordless motor (Cat.# Z359971) and blue polypropylene (autoclavable) (Cat.# Z359947).

• Luminometer Optocomp I (MGM Instruments).

• Epifluorescence Lumar V12 stereomicroscope equipped with a digital camera (AxioCam MRm) (Zeiss).

### Procedure

#### Microinjection

**1- **Prepare the microinjection mix containing 20 ng/μl of firefly luciferase and 2 ng/μl *Renilla *reporter plasmids. Add Buffer Tango 10× to a final concentration of 0.5×, and phenol red solution to a final concentration of 0.05%. When injecting morpholinos, they should be prepared to a final concentration of 1–10 μM.

**CAUTION! **When microinjecting exogenous molecules, such as PAMPs or drugs, check different *Renilla *reporter plasmids and select those with consistent expression.

**2- **Immediately after spawning, collect fertilized egg with a Pasteur pipette and place them in egg water on an agarose-modified Petri dish be means of the forceps.

**Figure 4:**
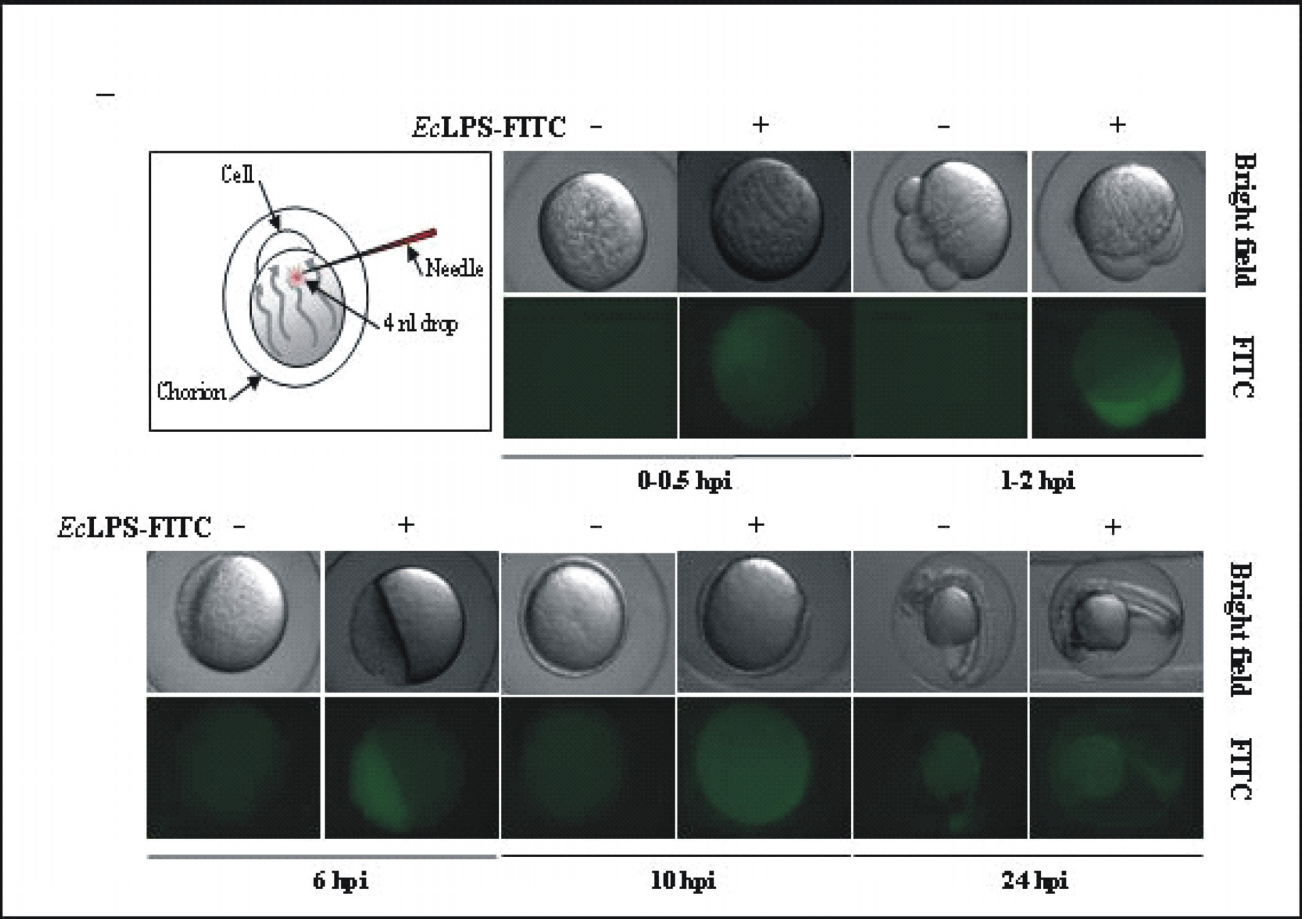
**Molecules microinjected in the yolk sac are rapidly delivered into the embryo**. **(A) **Schematic representation of the site of injection in one-cell zebrafish embryo. **(B) **Visualization of FITC labelled *Ec*LPS upon microinjection into the yolk sac. Embryos were microinjected with 3.5 ng LPS from *E. coli *strains 0111:B4 conjugated with FITC and examined at the indicated times with a LUMAR stereomicroscope. hpi, hours post-injection.

**CRITICAL STEP **It is very important to microinject the zebrafish embryos at early developmental stages (from one- to eight-cells) to ensure that the cytoplasmic flows introduce DNA into the cell (Fig. 4)

**3- **Load glass capillaries with 1–5 μl of microinjection mix by using a 0.5–10-μl microloader tip.

**4- **Put the Petri dish with the fertilized eggs under a stereomicroscope at 40× magnifications and place the loaded needle toward the yolk sac, close to the embryo cell. By using the microinjector, insert the tip of the needle into the yolk and inject a 4 nl drop by setting the proper pressure (50–60 Psi) and time (10–100 ms).

**5- **Incubate the injected embryos for 24–48 h at 28.5°C in egg water.

#### Quantitative evaluation of the response with the Dual-Luciferase Reporter Assay System

**6- **The next day, collect live injected embryos and put three of them in a 1.5 ml microcentrifuge tube.

**7- **Remove the egg water, wash the embryos with PBS and remove it completely.

**CAUTION! **Take care during the wash because the eggs may break.

**8- **Incubate the embryos with 50 μl of passive lysis buffer (PLB) 1× for 30 min at room temperature, shaking at 150 rpm.

**9- **After incubation, homogenize the embryos with the pellet pestle.

**CRITICAL STEP **From this point onwards, protect samples from light.

**10- **Spin the embryo extracts for 3 min at 13,000 rpm to remove cellular debris.

**11- **Measure firefly and *Renilla *luciferase activities according to the manufacturer.

• **Timing**

• Step 1: 30 min. Preparation of microinjection mix.

• Step 2: 10 min. Preparation for injection of 100 zebrafish embryos.

• Steps 3–5: 15 min. Microinjection of 100 zebrafish embryos.

• Steps 6–11: 2 h. Quantitative evaluation of the response of 100 zebrafish embryos with the Dual-Luciferase Reporter Assay System.

## Authors' contributions

F.A-P., M.L.C and V.M. designed the research, analyzed the data and wrote the paper; F.A-P. performed the research. All authors read and approved the final manuscript.
